# Dental Implants in Patients with Functional Diversity Under a Strict Prevention Protocol: A Retrospective Pilot Study

**DOI:** 10.3390/jcm15145667

**Published:** 2026-07-20

**Authors:** Javier Silvestre-Rangil, Santiago Isern-Hernández, Tamara Orpegui-Sánchez, Victoria Martínez-Mihi, Francisco Javier Silvestre, Cecilia Fabiana Márquez-Arrico

**Affiliations:** 1 Department of Stomatology, University of Valencia, 46010 Valencia, Spain; marmivic@uv.es (V.M.-M.); francisco.silvestre@uv.es (F.J.S.); cecilia.marquez@uv.es (C.F.M.-A.); 2 Private Practice, 46010 Valencia, Spain; santi.isern11@gmail.com (S.I.-H.); orpeguitamara@gmail.com (T.O.-S.)

**Keywords:** functional diversity, dental implants, implants success, marginal bone loss, prevention

## Abstract

**Background:** The aim of this study was to analyze the success of dental implants in patients with functional diversity by comparing success rates and marginal bone loss with those observed in patients without functional diversity, with both groups being subjected to a strict implant maintenance and hygiene protocol. **Methods:** A retrospective observational case–control study was designed. Two main study groups were established: one comprising patients with functional diversity (case group) and another including patients without functional diversity (control group), all of whom had received dental implants. Implant success rates were evaluated 12 months after prosthetic loading. Marginal bone loss was quantified according to the classification proposed by Lagervall and Jansson, later modified and validated by Corcuera-Flores et al. In addition, the Silness and Löe plaque index, presence of bruxism, implant characteristics, and rehabilitation techniques were recorded at each follow-up visit. **Results:** A total of 63 dental implants were placed in 20 patients included in the present study. The case group consisted of 9 patients who received 31 implants, whereas the control group comprised 11 patients who received 32 implants. No statistically significant differences were observed between groups in terms of implant success and MBL. Oral hygiene was assessed showed a statistically significant difference between groups (*p* = 0.001). Significant differences were also found regarding bruxism prevalence between groups, which was higher in the case group. **Conclusions:** The results obtained in the present study indicate that implant therapy in patients with functional diversity can achieve success rates comparable to those observed in patients without disabilities despite the higher prevalence of bruxism and a greater amount of dental plaque. These findings reinforce the importance of individualized treatment planning, adequate oral hygiene control, and periodic clinical follow-up in order to ensure long-term implant stability and peri-implant health.

## 1. Introduction

Individuals with functional diversity, including those with physical, intellectual, sensory, or multiple disabilities, experience a disproportionately high burden of oral diseases compared with the general population. The most prevalent oral health conditions in this population include dental caries, periodontal disease, dental trauma, and progressive tooth deterioration. Notably, Decayed, Missing, and Filled Teeth (DMFT) index values exceeding 20 have been reported, reflecting substantial unmet oral health needs and a significant cumulative burden of disease [1,2]. This increased burden of disease results from the interaction of multiple factors, such as limitations in performing adequate oral hygiene, the chronic use of certain medications, diets with high cariogenic potential, and the presence of economic, professional, and social barriers that hinder access to adapted dental care [3]. The consequences of the high prevalence of dental pathology together with barriers to early dental care increase the need for restorative and rehabilitative treatments [4].

Oral implantology is a continuously evolving field and may represent a viable therapeutic option for individuals with functional diversity, enabling the rehabilitation of oral function, improving aesthetic outcomes, and consequently enhancing health-related quality of life [5]. Nevertheless, due to the clinical and behavioral characteristics of this population, implant therapy is less frequently indicated and performed than in the general population, resulting in limited scientific evidence on the subject. Evidence regarding dental implant therapy in patients with disabilities remains scarce, with the existing literature largely consisting of isolated case reports and small case series, which precludes a comprehensive evaluation of long-term treatment outcomes [6,7,8,9,10,11,12]. Although some studies have included larger samples and longer follow-up periods, they still present methodological limitations, such as the absence of control groups and the heterogeneity of the study populations. The study involving the largest number of patients with disabilities rehabilitated with dental implants was conducted by Ekfeldt et al. [13], in which 88 implants were placed in 27 patients with a follow-up period ranging from 5 to 10 years. The authors concluded that implant therapy in patients with neurological disabilities can achieve satisfactory outcomes, with an 85% success rate.

Furthermore, studies focusing on specific diagnoses, such as Down syndrome, have also been published. In 2016, a retrospective series including 25 patients with this condition reported a failure rate of 23.2% among a total of 73 implants placed [14]. In 2017, the first case–control study involving patients with disabilities was published, comparing a control group with patients diagnosed with Down syndrome and cerebral palsy. In the Down syndrome group, 31 implants were placed, of which 29% failed during the four-year follow-up period. Furthermore, all implants exhibited some degree of marginal bone loss (MBL). In contrast, among the 71 implants placed in patients with cerebral palsy, 36% showed no MBL and no implant failures were recorded [15].

Dental implant maintenance and oral hygiene represent another important area that remains poorly investigated, with no studies to date having examined their potential association with implant success rates in people with disabilities. Nevertheless, current evidence in the general population suggests that proper home oral hygiene is one of the most important factors in maintaining peri-implant health. Effective biofilm control significantly reduces the risk of peri-implant mucositis and its progression to peri-implantitis, while patients with better oral hygiene habits demonstrate higher long-term implant survival rates and greater peri-implant tissue stability. Although the combined use of manual or powered toothbrushes, interdental brushes, and oral irrigators may improve plaque control, the evidence primarily supports the importance of effective mechanical biofilm removal rather than the superiority of any specific oral hygiene device. These findings highlight the need for future research to determine whether oral hygiene practices similarly influence implant outcomes in individuals with disabilities [16].

Finally, rehabilitation of masticatory function through dental implants has been associated with significant improvements in quality of life, aesthetics, and patient self-esteem. Therefore, the aim of this study was to assess the long-term success of dental implants in patients with disabilities by comparing implant osseointegration rates and MBL with those of patients without disabilities, under a standardized and strict implant maintenance and oral hygiene protocol.

## 2. Materials and Methods

### 2.1. Study Design

A retrospective observational case–control study was conducted at the Special Needs Unit of the Dental Clinic of the Fundació Lluís Alcanyís, Universitat de València. The study population comprised two groups: a case group consisting of patients with functional diversity who had received dental implants and a control group including patients without functional diversity who had undergone the same treatment. The study was conducted in accordance with the ethical principles of the Declaration of Helsinki and was approved by the Ethics Committee of the Universitat de València (reference No. 2025-ODON-3916816).

### 2.2. Study Participants

The study included patients over 18 years of age who had been rehabilitated with dental implants at the Dental Clinic for Patients with Special Needs of the Fundació Lluís Alcanyís, Universitat de València (*n* = 20 patients; *n* = 63 implants). The study group comprised patients with functional diversity (*n* = 9), whereas the control group included patients without functional diversity (*n* = 11). Only patients who had received oral hygiene instruction and were enrolled in a preventive maintenance protocol, with follow-up visits every 4 months for a minimum period of 24 months, were included.

Patients classified as ASA IV (patient has incapacitating disease that is a constant threat to life); those presenting acute infections at the implant placement sites; smokers; patients receiving treatments potentially affecting bone metabolism, such as long-term corticosteroids, bisphosphonates, or monoclonal antibodies; patients with active periodontal disease at the time of implant placement; and patients with systemic conditions that could interfere with osseointegration (endocrine, rheumatologic diseases, osteoporosis, chronic kidney and liver disease, immunodeficiency disorders, autoimmune diseases, anemia and malnutrition) were excluded.

### 2.3. Sample Size Calculation

For the present pilot study, the significance level was set at α = 0.05 (two-sided significance level, *p* < 0.05), assuming a maximum 5% probability of incorrectly rejecting the null hypothesis and concluding that a marginal bone loss of 1.0 mm exists when it does not (Type I error).

Given the pilot nature of the study, the sample size was determined based on feasibility and practical considerations, resulting in a total of 30 dental implants. According to methodological recommendations for pilot studies in dental research, this sample size is considered adequate to provide a reliable estimate of the standard deviation and variability of peri-implant marginal bone loss. Furthermore, it allows an accurate sample size calculation for the design of future adequately powered confirmatory clinical trials [17,18].

### 2.4. Study Protocol

The described protocol corresponds to the standard protocol for implant treatments in our unit, and was performed prior to the design of the study. After obtaining a complete medical history and performing a clinical examination, photographic records were obtained and cone-beam computed tomography (CBCT) scans were performed for all patients eligible for the study. Treatment planning was carried out by the same experienced clinician in implant-supported rehabilitations, who also placed all implants.

All included patients underwent periodontal treatment and both patients and/or caregivers received oral hygiene instructions, including tooth brushing, dental floss, superfloss, and interdental brushes. Patients received non-surgical periodontal treatment based on their needs, ranging from dental prophylaxis to scaling and root planing. Following enrollment, all participants were placed on a structured preventive maintenance program and attended follow-up visits at 4-month intervals throughout the study period.

All patients performed preoperative mouth rinses with 0.2% chlorhexidine, and 1000 mg of amoxicillin was prescribed one hour before surgery. Subsequently, a sterile surgical field was established and 4% articaine with epinephrine 1:100,000 was administered.

The surgical approach consisted of the conventional technique using paracrestal incisions without releasing incisions, performed with a No. 3 scalpel handle and a 15C blade directed toward the palatal area to preserve the maximum amount of keratinized buccal gingiva. Full-thickness flaps were elevated and the implant bed was prepared using a low-speed contra-angle handpiece with torque control and continuous external irrigation, following the drilling protocol for Straumann Standard Plus (SP) and Bone Level Tapered (BLT) implants with SLAactive surface (Straumann Group, Basel, Switzerland), under sterile saline irrigation to prevent bone overheating. Subsequently, all implants were inserted with a torque ranging between 30 and 50 Ncm. The final stage of the surgical procedure consisted of flap closure with 4/0 Supramid sutures.

Implant stability was evaluated between 8 and 12 weeks after implant placement, and prosthetic rehabilitation was initiated once implant stability had been confirmed. All the prosthetic restorations were performed by the same clinician.

Prosthetic rehabilitation was individualized for each patient according to their specific prosthetic requirements. In all cases, a fully digital workflow was implemented to minimize potential sources of error, using an intraoral scanner Trios 3 (3shape, Copenhague, Denmark) for digital impression acquisition. Prosthetic planning and fabrication were based on digital design and virtual models, with restorations manufactured using X ceramic materials and intermediate abutments, as appropriate. All prosthetic components were fabricated by the same dental laboratory to ensure standardization of the manufacturing process.

### 2.5. Studied Variables

Implant success was assessed 12 months after prosthetic loading according to the clinical and radiographic criteria established by Buser (the implant is clinically immobile upon examination; absence of pain, infection, neuropathy, paresthesia, or violation of the mandibular canal; absence of continuous peri-implant radiolucency on follow-up radiographs, marginal vertical bone loss of <1.5 mm during the first year of functional loading, followed by an annual bone loss of ≤0.2 mm thereafter, the implant provides satisfactory prosthodontic function without major mechanical complications) [19].

MBL was evaluated using panoramic radiographs (OPG) obtained for all participants at the time of prosthetic loading and annually thereafter. All radiographs were acquired using a Vatech Pax-i version 2.2 system with 1:1 magnification (Vatech, Hwaseong, Republic of Korea).

MBL was measured according to the classification proposed by Lagervall and Jansson for the diagnosis of marginal attachment loss around dental implants [20], later modified and validated by Corcuera-Flores et al. [21]. This method classifies dental implants into four groups according to MBL:

- Grade 0: dental implants without marginal bone loss.
- Grade 1: marginal bone loss involving one third or less than one third of the total implant length.
- Grade 2: marginal bone loss greater than one third but less than two thirds of the total implant length.
- Grade 3: marginal bone loss greater than two thirds of the total implant length.
- Grade 4: implant loss.

At each follow-up visit, the Silness and Löe Plaque Index [22], the presence of bruxism, implant characteristics, and prosthetic rehabilitation parameters were systematically recorded.

### 2.6. Statistical Analysis

Statistical analyses were performed using IBM SPSS Statistics version 29 (IBM Corp., Armonk, NY, USA). Descriptive statistics were calculated for all study variables, and comparative analyses were conducted between groups. Statistical significance was set at *p <* 0.05. Data distribution was assessed using the Kolmogorov–Smirnov and Shapiro–Wilk tests, which indicated a non-normal distribution. Consequently, non-parametric analyses were performed using the Mann–Whitney U test for continuous variables. Categorical variables, including sex distribution, were compared using the Chi-square test.

## 3. Results

A total of 63 dental implants were placed in 20 patients included in the present study. The case group comprised 9 patients who received 31 implants, whereas the control group comprised 11 patients who received 32 implants (Table 1). No significant differences were observed between groups regarding age (Mann–Whitney, *p* = 1.000) and sex distribution (Chi-square, *p* = 0.527), indicating baseline comparability. Within the case group, there were two patients diagnosed with autism spectrum disorder, two with Down syndrome, two with intellectual disability, one with Schizophrenia, one with Williams syndrome, and one with cerebral palsy.

Table 2 summarizes the statistical analysis of the study variables. Follow-up duration was compared between groups using the Mann–Whitney U test. Although the mean follow-up period was longer in the control group (7.10 ± 3.55 years; range, 2–17 years) than in the case group (5.00 ± 2.15 years; range, 2–11 years), the difference was not statistically significant (*p* = 0.054).

Implant success rates were 100% in the case group and 96.9% in the control group. Only one implant failure due to lack of osseointegration was recorded in the control group. No statistically significant differences were observed between groups (*p* = 0.329).

Regarding MBL, in the case group, 27 implants (87.1%) corresponded to grade 0 of the Lagervall and Jansson scale modified by Corcuera-Flores et al., 3 to grade 1 (9.7%), and one to grade 2 (3.2%). In the control group, 30 implants (93.8%) showed no bone loss, 1 to grade 1 (3.1%), and there was one implant loss before osseointegration (3.1%). No statistically significant differences were observed between groups (Mann–Whitney U test, *p* = 0.305). In the case group, MBL ranged from 0 to 2 mm, with a mean value of 0.13 ± 0.43 mm, whereas in the control group, MBL also ranged from 0 to 2 mm, with a mean value of 0.07 ± 0.37 mm.

Analysis of the distribution of MBL categories revealed that 90.0% of implants in the case group and 96.8% of implants in the control group exhibited no detectable MBL. However, the difference between groups was not statistically significant (Chi-square test, *p* = 0.285).

Oral hygiene, assessed using the Silness and Löe Plaque Index, differed significantly between groups (Mann–Whitney U test, *p* = 0.001). The mean plaque index score was higher in the case group (2.00 ± 0.95; range, 0–3) than in the control group (1.10 ± 0.82; range, 0–2).

The prevalence of bruxism was also significantly higher in the case group than in the control group (90.3% vs. 59.4%, respectively; Chi-square test, *p* = 0.014).

Implant length was analyzed using the Mann–Whitney U test and showed similar values in both groups. In the case group, implant length ranged from 6 to 12 mm, with a mean value of 9.93 ± 1.230 mm. In the control group, implant length ranged from 8 to 12 mm, with a mean value of 9.79 ± 0.819 mm, with no statistically significant differences between groups (*p* = 0.453).

Regarding implant design, cylindrical implants were used in 80.6% of implants in the case group and 67.7% of implants in the control group. No statistically significant differences were observed between groups (Chi-square test, *p* = 0.246).

Regarding the distribution according to the prostheses used, 17 of the implants in the case group were restored with single crowns compared to 21 in the control group. In the case group, 2 implants were restored with a bridge. The remaining implants, 12 in the case group and 10 in the control group, were included in restorations with hybrid prostheses. With respect to prosthetic rehabilitation, analysis using the Chi-square test demonstrated that single crowns were the most frequently used prosthetic restoration in both groups, although implant-supported hybrid prostheses and overdentures were also included. No statistically significant differences were observed in this variable either (*p* = 0.272), suggesting a homogeneous distribution of the prosthetic solutions applied.

## 4. Discussion

The aim of the present study was to evaluate the feasibility and clinical outcomes of implant therapy in patients with functional diversity by comparing them with a control group of patients without disabilities. After analyzing the collected data, no statistically significant differences were observed in either the success rate of dental implants or MBL between groups. These findings support the hypothesis that implant-supported rehabilitation is a viable and predictable therapeutic option for patients with functional diversity, provided that appropriate treatment planning and rigorous clinical follow-up are maintained, which is consistent with previously published literature on the subject [6,7,8,9,10,11,12,13].

One of the most relevant aspects in evaluating the success of dental implants is the assessment of peri-implant health. Currently, peri-implant disease has become a major focus of research due to its impact on long-term implant survival [23]. Its diagnosis is based on a combination of clinical parameters, among which MBL is considered one of the most sensitive and objective indicators for assessing peri-implant tissue status [24]. The results obtained in the present study showed no statistically significant differences between groups. These values were clearly below the clinically acceptable threshold, established as up to 1.5 mm during the first year after implant placement and approximately 0.2 mm annually thereafter. This bone stability suggests that, despite the potential clinical difficulties associated with functional diversity, dental implants exhibit biological response comparable to that observed in patients without disabilities [19].

Despite following the same protocol between groups, daily oral hygiene for patients with functional diversity continues to be a challenge for both the patients themselves and their caregivers. In the present study, a higher prevalence of bruxism and poorer oral hygiene levels were observed in the group of patients with functional diversity. A recent investigation involving 148 patients analyzed the possible relationship between bruxism and periodontal diseases, based on the worsening of oral hygiene due to joint pain and the overload of the periodontal ligament as an exacerbating factor for bone loss [25]. The authors proposed that this relationship may be mediated by impaired oral hygiene secondary to pain and discomfort, together with excessive mechanical loading of the periodontal ligament, which may contribute to periodontal tissue breakdown and alveolar bone loss. After analyzing the results, no significant associations were found between bruxism and periodontal pathology, even when oral hygiene status was evaluated [25]. These findings are consistent with those observed in our study, since although a higher prevalence of bruxism and poorer oral hygiene were identified among patients with functional diversity, these variables did not translate into greater MBL or a higher implant failure rate, which may be related to the standardized treatment and maintenance protocol applied. It is currently well established that appropriate management of occlusal loading on implant-supported prostheses, together with adequate oral hygiene control, may help prevent bone loss and prosthetic complications such as crown fractures or decementation [26].

Regarding the radiographic methodology used, although periapical radiography has been reported to provide higher accuracy for MBL measurement, OPG was selected in this study. This choice was based on several clinical and practical considerations. First, OPG allows a panoramic view of both dental arches in a single image, facilitating the simultaneous evaluation of multiple implants. This feature is particularly useful in patients with extensive rehabilitations or limited cooperation, as frequently occurs in individuals with functional diversity. In addition, it is a rapid and less invasive technique compared with multiple periapical radiographs, thereby improving patient tolerance and allowing an overall assessment of bone status. It is also commonly used in epidemiological studies [27]. Although OPG presents lower resolution and may be affected by geometric distortions, its use is considered valid when standardized protocols are applied and technical consistency in image acquisition is maintained [28]. Furthermore, an important factor in this investigation is that all radiographs were obtained using the same imaging and positioning system, and measurements were performed by a single examiner to minimize potential bias. This technique has also been validated in previous studies [20,21].

Analysis of the results revealed a success rate of 100% in the group of patients with functional diversity and 96.9% in the control group. These results are consistent with those reported in previous studies conducted in similar populations [6,7,8,9,10,11,12,13,14,15,29].

Ekfeldt et al. [13] described an 85% survival rate in patients with neurological disorders after a follow-up period ranging from 5 to 10 years, reporting a higher incidence of surgical and maintenance complications. Similarly, Oczakir et al. [29] reported a 100% survival rate in loaded implants, although they highlighted difficulties related to oral hygiene, particularly in patients with Down syndrome. In the study conducted by López-Jiménez et al. [12], an implant loss rate close to 6% was observed, mainly concentrated among patients with genetic disorders. Finally, Corcuera-Flores et al. [15] identified important differences according to the type of disability, with a 29% implant loss rate in patients with Down syndrome and no failures in patients with cerebral palsy. In comparison with these studies, the results of the present work demonstrated a high success rate and minimal MBL, although in our pilot study, we only presented 2 cases Down syndrome [14,15]. These findings may be associated with the implementation of strict clinical protocols, appropriate patient selection, and regular follow-up focused on oral hygiene control and peri-implant maintenance.

A preventive approach implemented before and after surgery may have contributed to these favorable outcomes. This is in line with the literature indicating that periodontal maintenance and regular follow-up are key factors in preventing peri-implant diseases and marginal bone loss [30].

Preoperative medical evaluation allowed the identification of systemic diseases, habits, and pharmacological treatments that could interfere with the osseointegration process. It is currently known that factors such as osteoporosis, angiogenesis defects, and other metabolic disorders such as diabetes may affect implant osseointegration and subsequent bone remodeling [31]. Importantly, patients included in this study did not present systemic conditions that could have influenced bone remodeling, nor did they show metabolic disorders, thereby reducing the influence of confounding variables on MBL outcomes.

Similarly, periodontal status was assessed and the absence of active periodontal disease was confirmed before implant placement. This approach was based on recent findings linking shared pathogenic pathways between active periodontitis and peri-implantitis, thereby aiming to reduce the risk of complications and MBL [32].

**Figure 1:**
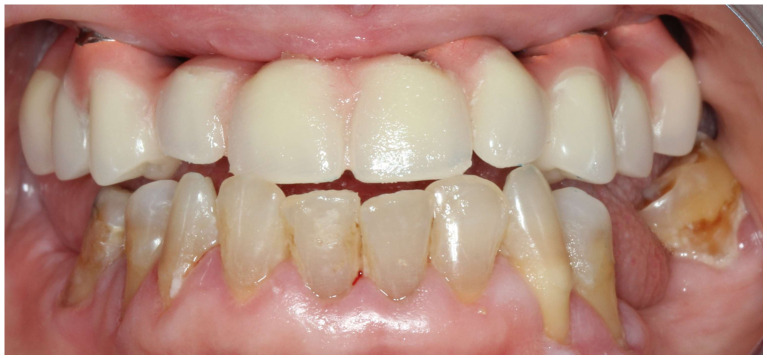
Hybrid prosthesis on implants with a hygienic design.

Another fundamental aspect was prosthetic design. Implant-supported prostheses were planned to facilitate proper oral hygiene, incorporating wide and accessible embrasures to allow cleaning with interdental brushes and other oral hygiene devices (Figure 1). This aspect is particularly relevant in patients with functional diversity, in whom cooperation may be limited and oral hygiene maintenance often depends on the support of caregivers or relatives.

Our findings reinforce those reported by Bogner et al. in 2025 [33], whose 14-year follow-up cohort demonstrated implant-supported prosthesis survival rates of approximately 86% in patients with cognitive and functional disabilities after applying a combination of appropriate prosthetic design and a preventive strategy based on strict oral hygiene maintenance with caregiver and family involvement. In line with these authors, we intend with this pilot study, and subsequent research in the field, that these patients should not be systematically excluded from implant treatments solely because of their medical condition or disability, but that the indication should be based on an individualized assessment of the benefit-risk balance.

In this regard, previous studies further support the concept that systemic conditions may not represent the predominant determinant in the etiology of MBL. Notably, El-Sawy et al. demonstrated that, among patients with type 2 diabetes mellitus, oral hygiene status had a greater influence on peri-implant bone loss than glycemic control, underscoring the predominant role of local factors [34,35]. Current evidence suggests that most systemic conditions, including osteoporosis, oral lichen planus, rheumatoid arthritis, HIV/AIDS, Crohn’s disease, Sjögren’s syndrome, immunosuppressive status, and ectodermal dysplasia, do not appear to significantly compromise dental implant outcomes, although the available evidence is generally limited by small sample sizes, short follow-up periods, and methodological heterogeneity [35].

Previous studies have demonstrated that fully guided implant placement protocols can improve the accuracy of implant positioning during the rehabilitation of edentulous maxillary arches [36,37,38]. Enhanced positional accuracy may facilitate prosthetically driven implant placement and contribute to more predictable restorative outcomes, particularly in anatomically complex cases or full-arch rehabilitations. Nevertheless, clinicians should balance these potential advantages against practical considerations, including the additional costs, workflow complexity, learning curve, and the need for dedicated digital equipment [32]. Consequently, the decision to implement fully guided surgery should be based on the specific clinical scenario and the anticipated benefits for each patient.

Although fully guided implant surgery was not employed in the present study, a digital workflow was systematically incorporated during the prosthetic phase. Digital intraoral scanning, computer-aided design, and standardized laboratory procedures were used to optimize the fit of implant-supported restorations, both single-unit and multiple-unit prostheses [32]. This digital restorative workflow, focused exclusively on the prosthetic phase of treatment, was based on intraoral scanning for implant position recording to facilitate the design and fabrication of the definitive restorations. Passive fit was clinically verified through a prosthetic passivity test before definitive delivery to ensure an accurate prosthetic fit. Furthermore, the prostheses were designed in close collaboration with the dental laboratory, with particular attention to providing appropriate contours and access for effective oral hygiene. This workflow was intended to minimize cumulative errors throughout the restorative process, thereby contributing to the predictability, reproducibility, and long-term success of the final prosthetic outcomes.

Despite the positive outcomes obtained, this study presents certain limitations that should be considered. First, the small sample size may limit the generalizability of the results. The follow-up imbalance between groups could contribute to the risk of bias. Second, functional diversity was considered as a broad category encompassing different physical, intellectual, and sensory conditions, which may have masked specific differences between subgroups. Third, although clinically justified, the use of OPG for MBL measurement provides lower accuracy than periapical radiographs. Finally, the follow-up period may still be insufficient to fully evaluate the very long-term evolution of dental implants. Nevertheless, the fact that all surgical procedures were performed by a single experienced surgeon, together with comprehensive medical history assessment, periodontal evaluation, strict follow-up, and rigorous oral hygiene control, strengthens the reliability of our findings.

Future research should include larger sample sizes and perform differentiated analyses according to the specific type of functional diversity. In addition, longitudinal studies with longer follow-up periods and the incorporation of more precise radiographic techniques, such as standardized periapical radiographs or CBCT, are recommended to improve the accuracy of bone measurements.

## 5. Conclusions

In conclusion, the results obtained in the present study indicate that implant therapy in patients with functional diversity can achieve success rates comparable to those observed in patients without disabilities. These findings reinforce the importance of individualized treatment planning, adequate oral hygiene control, and periodic clinical follow-up in order to ensure long-term implant stability and peri-implant health.

The findings of this study reinforce the concept that appropriate patient selection and a comprehensive preventive protocol contribute to the success of dental implants, even in the presence of challenges such as a higher prevalence of bruxism and increased dental plaque accumulation during maintenance visits.

## Figures and Tables

**Table 1:** Baseline characteristics of the study sample. * One implant failed during follow-up.

Group	Male, *n* (%)	Female, *n* (%)	Patients/Implants, *n*
**Cases**	6 (66.7%)/17 implants	3 (33.3%)/14 implants	9/31
**Controls**	5 (45.5%)/15 implants	6 (54.5%)/17 implants *	11/32
**Total**	11	9	20/63

**Table 2:** Statistical comparison between the variables studied. * Statistically significant differences (*p* < 0.05). Data are presented as mean ± SD (range) or *n* (%). IQR: interquartile range.

Variable	Case Group (*n* = 31 Implants)	Control Group (*n* = 32 Implants)	Statistical Test	*p*-Value
Age (years)	Median: 49.0	Median: 46.0	Mann–Whitney	1.000
	IQR: 18	IQR: 20.0		
	Range: 33–61	Range: 30–69		
Sex (male) (%)	54.8 ± 0.0909	46.9 ± 0.0911	Chi-square	0.527
Follow-up time (years)	Median: 5	Median: 8	Mann–Whitney	0.054
	IQR: 2	IQR: 6		
	Range: 2–11	Range: 2–17		
Success rate (%)	100	96.9 ± 0.0316	Chi-square	0.329
Marginal bone loss (mm)	Median: 0.0	Median: 0.0	Mann–Whitney	0.305
	IQR: 0	IQR: 0		
	Range: 0–2	Range: 0–2		
No bone loss (%)	90 ± 0.0548	96.8 ± 0.0321	Chi-square	0.285
Oral hygiene (Silness and Löe)	Range: 0–3; 2.00 ± 0.947	Range: 0–2; 1.10 ± 0.817	Chi-square	0.001 *
Probable bruxism (%)	90.3 ± 0.054	59.4 ± 0.0854	Chi-square	0.014 *
Implant length (mm)	Median: 10	Median: 10	Mann–Whitney	0.453
	IQR: 0	IQR: 0		
	Range: 6–12	Range: 8–12		
Implant design (cylindrical) (%)	80.6 ± 0.0722	67.7 ± 0.0854	Chi-square	0.246
Prosthesis type (crown) (%)	54.8 ± 0.0909	67.7 ± 0.0854	Chi-square	0.272

## Data Availability

No new data were created or analyzed in this study. Data sharing is not applicable to this article.
